# Visual discrimination training increases the speed stimulus processing and leads to an earlier onset of stimulus encoding

**DOI:** 10.1371/journal.pone.0330284

**Published:** 2025-08-18

**Authors:** Camila Bustos, Rodrigo Montefusco-Siegmund, Fernando Peña, María de la Luz Aylwin

**Affiliations:** 1 Laboratorio de Neurofisiología, Facultad de Medicina, Universidad de Talca, Talca, Chile; 2 Human Cognitive Neurophysiology and Behavior Lab, Facultad de Medicina, Universidad Austral de Chile, Valdivia, Chile; 3 Centro de Estudios Interdisciplinarios del Sistema Nervioso, Facultad de Medicina, Universidad Austral de Chile, Valdivia, Chile; 4 Independent Researcher, Halifax, Nova Scotia, Canada; 5 Centro de Investigación en Ciencias Cognitivas (CICC), Facultad de Psicología, Universidad de Talca, Talca, Chile; Kyoto University of Education: Kyoto Kyoiku Daigaku, JAPAN

## Abstract

Wide experience with complex visual stimuli results in better performance and faster responses in object discrimination, categorization, and identification through perceptual learning and expertise. Visual experts exhibit an earlier onset of the availability of stimulus information for encoding and a reduction of the encoding duration required for discrimination and individuation. However, it is still unresolved whether perceptual learning and expertise shapes the speed of perceptual processing in the first milliseconds after stimulus onset. Twenty seven participants developed perceptual learning and expertise through discrimination of pairs of Kanji stimuli across six sessions. Discrimination sensitivity was evaluated at four training levels with encoding durations between 17 and 1000 ms. Behavioral results show a gradual increase in sensitivity and a reduction in encoding duration required for a given performance with discrimination training. A shifted exponential function fitted to the sensitivity data revealed that training leads to a faster rate of performance change with encoding durations, suggesting increases in the speed of information extraction, as well as an earlier availability of stimulus information for encoding, suggesting an earlier onset of information extraction. Interestingly, the increase in the rate of performance paralleled that of sensitivity with training, suggesting an association with perceptual learning and expertise. Besides, the earlier availability of stimulus information is achieved after two training sessions, likely reflecting the acquisition of stimuli familiarity. The faster speed of information extraction and the earlier stimulus information extraction for encoding, likely contribute to faster responses and higher performance, typical of perceptual experts in object discrimination and individuation. These findings provide additional evidence for the outcome of discrimination training on stimulus processing in the first milliseconds after stimulus onset.

## Introduction

In our daily activities we discriminate, categorize and recognize objects instantly. Extensive experience with a category of stimuli leads to perceptual learning and expertise [1] characterized by a high speed and accuracy of responses in discrimination, categorization and individuation tasks. Visual perceptual experts can correctly discriminate both familiar and unfamiliar stimuli from the expertise category, such as cytopathological images [2,3], X-Rays [4,5] and fingerprints [6]. Perceptual expertise is achieved through perceptual learning by exposure in natural conditions [7–9] or extensive training with a stimuli category [10–16]. Although visual expertise enhances early stimulus processing [17], it is yet unknown how early stimulus processing is modulated during perceptual learning and the acquisition of perceptual expertise. In the present study, we assessed the modulation of early visual processing while participants develop perceptual learning and expertise by discriminating Kanji characters.

Early studies in visual perceptual learning and perceptual expertise in natural and lab trained experts (reviewed by [1,16,18–20]) targeted performance and speed of responses in different tasks but only a few examined the modulation of early processing after stimulus onset. One study on texture detection of 10 ms stimuli with encoding times from 20 to 300 ms, showed a gradual increase in performance, a reduction of stimulus processing time and an earlier onset of performance greater than chance [21]. Besides, visual experts exhibit an earlier onset of availability of stimulus information, defined as the minimum stimulus encoding duration for performance greater than chance, but an unchanged rate of stimulus information extraction [17].

A distinctive attribute of perceptual learning and expertise is the transfer of learning to novel stimuli [7,22]. Perceptual experts generalize performance to all objects within the expertise category [8,14,23–27] indicating the development of implicit pattern recognition and selective information extraction skills [16] that promote stimulus discriminability for the trained category [28] and a minor role of recognition memory [29].

Visual perceptual learning and expertise is based on learning induced changes in perceptual and decisional processes [25,30–33], but the exact location of the plasticity has been debated as evidence has been provided for early and late stages of cortical processing [34–36]. Early research in perceptual learning and expertise proposed a mechanism of differentiation learning for the discovery of distinctive features along a dimension required to distinguish the objects [18]. Later studies on perceptual learning of simple features [37–39] proposed two theories for the neural changes underlying learning, early cortical stages or higher areas by the reweighting of the connectivity form early cortical areas [36], An integrated reweighting theory of perceptual learning). In contrast, studies on perceptual learning and expertise acquisition of complex stimuli show increased activation and changes in the distribution of activity of object-specific visual areas [22,40], characteristic of perceptual expertise. In summary, visual perceptual learning and expertise enhances early stimulus processing [17]. However, the pathway of early processing modulation as a function of the levels of perceptual learning and expertise, specifically the rate of information extraction and the onset of stimulus information extraction for encoding, remains unresolved.

Here, we used a new approach to evaluate early stimulus processing during perceptual learning and the acquisition of expertise. We obtained the rate of stimulus information extraction after the onset of stimulus and the onset of the stimulus information extraction for encoding while participants develop visual perceptual learning and expertise over six discrimination training sessions with Kanji stimuli in a group of Spanish-speaking participants. The impact of training on stimulus processing in the few milliseconds after stimulus onset was assessed as the sensitivity with several brief encoding durations in four interleaved evaluation sessions. We hypothesized that discrimination training would promote faster speed of information extraction after stimulus onset evaluated as a higher rate of performance change with increasing encoding durations and an earlier onset of stimulus information available for encoding, evaluated as an earlier onset of performance above chance.

## Methods

### Participants

Twenty seven right-handed participants (age, M = 20.9, SD = 2.86, 13 female), with normal or corrected-to-normal vision, participated in this study in exchange for monetary compensation (approximately $32 US dollars). All participants, college or graduate students recruited through advertisements placed in the University of Talca, gave written informed consent. The behavioral measurements were conducted in accordance with Protocol # 036–2021, approved by the Ethical Committee of the University of Talca. The recruitment period was from January 3^th^, 2022 to June 9^th^, 2023.

### Stimuli

Stimuli were forty five exemplars of Kanji characters of 17–18 traces obtained from the Microsoft Office tool for Japanese writing. We selected the stimuli based on the work of Chen et al. [41] in holistic processing and our previous studies [11,42] to develop lab trained perceptual learning and perceptual expertise in participants performing discrimination training. The Kanji stimuli were selected to assess discrimination based on their visual features, not their orthographic or linguistic features. Spanish is the primary language of all participants, and they had no prior experience with the stimulus category, as specified in the recruitment interview.

Stimuli (1.5 x 1.5 visual degrees) were presented with MATLAB (Mathworks, Natick, MA) and the Psychophysics Toolbox on a black background at the center of a 23 inch monitor (ASUS Designo MX239H Monitor, refresh rate 60 Hz) at a distance of 57 cm.

### Procedure

**Fig 1:**
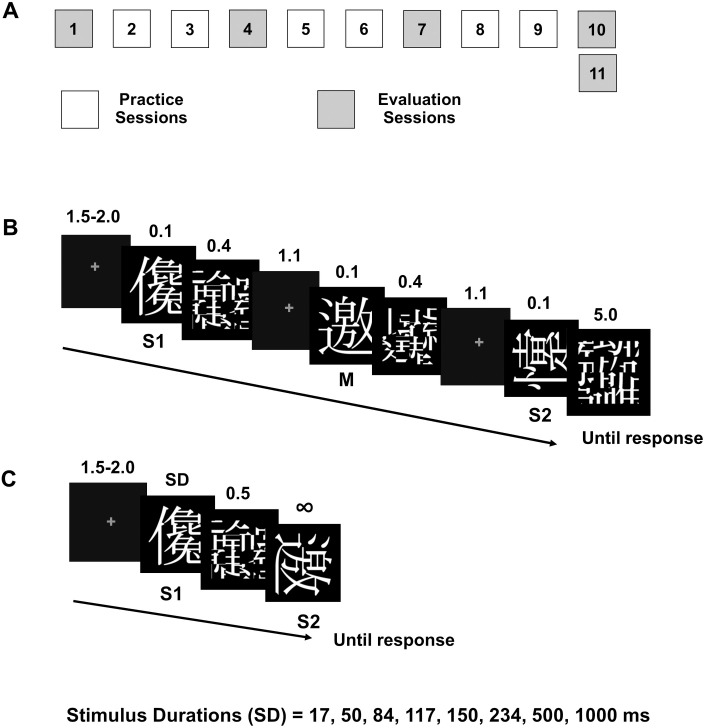
Experimental and trial sequence in training and evaluation/generalization sessions. A. Sequence of sessions, six training sessions (2, 3, 5, 6, 8 and 9), four evaluation sessions (1, 4, 7, and 10) and the generalization session (11). B. An example of the sequence of events in a training trial consisting of a sequential matching task with backward masking. Participants were exposed to stimulus 1 (S1t, 0.1 s), perceptual mask (PM, 0.2 s) and stimulus 2 (S2t, 0.1 s), each followed by scrambled fragments of Kanji stimuli as backward masks (BM, 0.4 s). C. An example of an evaluation and generalization trial. Participants were exposed to stimulus 1 (S1e, variable duration presented in a pseudorandom order) and stimulus 2 (S2e, 0.1 s) and a backward mask (BM, 0.4 s) between S1e and S2e. The same Kanji exemplars were used in the training and evaluation sessions. In the generalization trial, the stimuli were novel exemplars of the Kanji category.

The experimental design involved a sequence of eleven sessions (Fig 1A). Participants performed six training sessions (2, 3, 5, 6, 8 and 9) to develop perceptual learning and expertise, four evaluation sessions (1, 4, 7, and 10) to assess the stimulus information extraction in the first milliseconds after stimulus onset, and a generalization session (11) to evaluate the transfer of learning to new exemplars of the Kanji category reflecting the generalization of learning for the stimuli category. In the following sections, we describe the procedures for the training, evaluation, and generalization sessions.

### Training sessions

Participants performed a same-different task described in detail previously [11,42] with pairs of initially unfamiliar Kanji stimuli [41]. We chose the sequential matching task for discrimination training to promote learning to differentiate stimuli at the individual level based on the relevant visual features or patterns. Previous evidence shows that training on visual discrimination of meaningless stimuli develops the ability to extract the relevant information for the differentiation of a category of stimuli [22] and the development of perceptual expertise [22,43]. We selected Kanji stimuli unknown to Spanish-speaking participants so that discrimination was based on visual features rather than orthographic or linguistic features.

Each trial, began with a fixation dot of variable duration (1.5 to 2.0 s), followed by a sequence of two stimuli S1t and S2t, each lasting 0.1 s (Fig 1B), sufficient for detection and individuation of complex stimuli in experts [17,44] and the acquisition of expert-like performance [11,42]. To reduce the effect of stimulus repetition on S2t, a perceptual mask (PM, 0.1s), a Kanji different from S1t and S2t, was presented in between S1t and S2t, as in shape discrimination training [22] and rapid serial presentation [45,46]. S1t and PM were each followed by a backward mask (BM, 0.4 s) built as scrambled fragments of the Kanji stimuli, to stop further retinal stimulation by the relevant stimuli [17,47,48]. S2t was followed by a BM that lasted until participant’s response, with a maximum of 5.0 s. The BM after S1t and PM there was an interval of 1.1 s where a dot was presented to facilitate the eye fixation on the screen, Thus, inter-stimulus intervals S1t-PM and PM-S2t were both 1.6 s [11].

Seated in a dimly light room, participants began each trial by pressing the center button of a seven button RB-740 Response Pad (Cedrus Corporation, San Pedro, CA, USA). After S2t, participants had to respond if the pair S1t-S2t was perceived as “*same*” or “*different*” by pressing either the first (left) or seventh (right) pad buttons as accurately as possible. Half of the participants responded “*same*” with the left button and “*different*” with the right button and the remaining half with the reverse order. To avoid discrimination based on the retinal matching of S1t-S2t same pairs and to promote object discrimination based on object features, S2t was rotated by 90 degrees either clockwise or counter-clockwise in a pseudo-random manner [11,49,50] in all trials. Participants were informed of this rotation.

We built six stimuli lists out of the pool of 30 Kanji exemplars (lists and Kanji stimuli available at https://data.mendeley.com/datasets/9fw34stcnz/1), each containing a random sequence of same and different pairs, with an equal frequency of each exemplar as S1t, S2t and PM and a 50/50 ratio of same and different pairs; all lists included the 30 exemplars. The order of the lists was randomly selected and counterbalanced between participants. Each session of 360 trails lasting approximately 50 minutes, was divided into 4 blocks of 90 trials each. Between blocks, participants were free to rest upon request.

Participants did not receive feedback on their performance. Because we predicted a high variability in motivation and attention during the task, all participants were encouraged to perform well at the beginning of each session. In addition, the sensitivity was estimated at the end of each session and if there was no increase in sensitivity, participants were instructed to make an effort to be more attentive and perform better at the beginning of the following session. No quantitative information regarding correct or incorrect responses was provided.

### Evaluation sessions

Participants performed a same-different task previously described [17]. Each trial began with a fixation dot of variable duration (1.5 to 2.0 s), followed by a sequence of two stimuli, S1e and S2e. S1e was followed by a BM (0.5 s) to halt further retinal stimulation (Fig 1C). S2e lasted until the participant’s response with a maximum of 5.0 s. S2e was rotated by 90 degrees either clockwise or counter-clockwise in a pseudo-random manner in all trials and participants were informed of this rotation. To assess the role of encoding duration on performance in the first milliseconds after the stimulus onset, S1e had one of the following durations 17, 50, 84, 117, 150, 234, 500 or 1000 ms, selected in a random manner. These durations are multiples of the monitor’s refresh rate (60 Hz). Thus, the time between the onset of S1e and S2e or stimulus onset asynchrony (SOA) is the duration of S1e from 17 to 1000 ms plus 0.5 s of the BM.

Seated in a dimly light room, participants began each trial by pressing the center button of a seven button response pad. We built four stimuli lists out of the same 30 Kanji exemplars presented in the training sessions (lists and Kanji stimuli available at https://data.mendeley.com/datasets/9fw34stcnz/1), each containing a random sequence of same and different pairs, with an equal frequency of each exemplar as S1e and S2e and a 50/50 ratio of same and different pairs. The order of the lists was randomly selected and counterbalanced between participants.

Each evaluation session of 480 trials lasting approximately 30 min, included 60 trials for each of the eight encoding durations. Of the 60 trials, 30 were “*same*” and 30 “*different* pairs. The “*same*” pairs were built from the 30 exemplars and the “*different*” pairs were a combination of the 30 exemplars, each exemplar was presented 4 times per encoding duration. Each session was divided in 6 blocks of 80 trials each. Between blocks, participants were free to rest upon request. Just like in training sessions, participants did not receive feedback on their performance and no quantitative information regarding correct or incorrect responses was provided.

### Generalization session

To evaluate the generalization of learning to the Kanji category, a characteristic of perceptual learning and expertise [1,39,43], participants performed the same task as in the evaluation sessions (Fig 1C) but with a set of novel Kanji stimuli. We built a list of 480 trials out of 15 Kanji exemplars different from those included in sessions one to ten. Of a total of 27 participants that completed the training sessions and evaluation sessions, 16 performed the generalization session because several participants were unable to attend session 11.

### Data analysis

We predefined a minimum criterion of **d’* *≥ 0.4 \\J-fs04\j-plos-l\Production\PONE\pone.0330284\OPT_XMLof performance improvement at the fifth training session (overall session 8, Fig 1A) for participant inclusion. Participants that did not meet an increase in performance of 0.4 between sessions 1 and 8 were eliminated before completing all sessions. Because there was no feedback on performance and there was a monetary incentive, we assumed that the eliminated participants were not solving the task. Based on this criterion, 27 participants completed the training and evaluation sessions and data analysis was carried out for these sessions. Moreover, the generalization session was performed in a subset of 16 participants, because as previously stated, they were not available to complete the last session. Since our experiments involved the participants coming to the lab for 10 consecutive days, excluding weekends, it was difficult to recruit participants and engage them for the complete set of sessions.

For the analysis we only included the trials with response times between 0.2 and 5.0 s. We calculated the sensitivity for each participant at each session using the signal detection theory, assuming the independent strategy with dprime_simple (https://github.com/kc13/dprime_simple/blob/master/dprime_simple.m) written in Matlab (MathWorks Inc., Natick, MA).

### Statistical analysis

Statistical differences in performance were estimated with one- or two-factor repeated measures ANOVA [17]. The within-subject effects were corrected with Greenhouse-Geisser for significant Mauchy spehricity tests. Differences between means were assessed with two-tailed paired t-tests and the difference between performance greater than chance and d prime = 0 was estimated by a two-tailed one sample t-test. Outcomes of t-tests were corrected for multiple comparisons with the Benjamini-Hochberg (BH) procedure considering a BH critical value of.05 (5% false discovery rate). We report the raw *p* values from the t-tests and indicate which of them are significant after BH correction. We assessed the normality of the data with the Shapiro-Wilk test (with 99% confidence). Except for the fitting, all values are reported as mean ± SD of the mean in the main text and as mean ± SEM in the Figures.

### Curve fitting

To estimate the onset of information availability and rate of performance change with encoding times, we fitted an exponential function to the mean sensitivity (*d’*) data with encoding durations at sessions 1, 4, 7 and 10. We obtained the intercept with the x axis, the encoding duration were performance is equal to chance (*d’* = 0), representing the lower limit for the onset of stimulus encoding. We also obtained the initial speed of performance change with brief encoding durations or the rate of stimulus information processing after stimulus onset; and performance asymptote or the maximum performance at each training level.

A shifted exponential function was fitted to the mean (*d’*) values representing the discrimination sensitivity at each time point:


$$d’=A\left(1-e^{-R(t-I)}\right)$$

for *t > I*, otherwise *d’ = 0*. *I* is the intercept, *R* is the rate of approach to asymptote, *A* is the asymptote, and *t* is *t*he stimulus encoding duration.

Curve fitting was implemented by solving nonlinear least squares using the Levenberg-Marquardt (nlsLM) optimization algorithm contained in the function of the R package minpack.lm (https://rdrr.io/cran/minpack.lm/, Elzhov et al. 2023). The range of values for the parameter starting points needed by the nlsLM, were limited to intercept, 10^−7^–500.0, growth rate 0.0001–10.0, asymptote, 1.0–10.0. Since *t* is measured in milliseconds, for computational purposes 10^−7^ is zero. We solved the nonlinear least squares for each session without restrictions in the parameters I, R and A. Once the algorithm converged into a stable solution, the 3 model parameters were found together, with an estimate of goodness-of-fit, using *R*^*2*^, defined as:


$$R^{2}=1-\frac{\sum_{i=1}^{N}{\left({d^{\prime }}_{i}-{\hat{d^{\prime }}}_{i}\right)}^{2}}{\sum_{i=1}^{N}{\left({d^{\prime }}_{i}-\overset{―}{d^{\prime }}\right)}^{2}}\;$$

where **d’*_*i*_* is the observed discrimination index at data point *i* (i* *= 1 happening at *t = *17 ms, *i =* N, happening at *t = *1000 ms), $\hat{d}$*’*_*i*_ is the value predicted by shifted exponential function at data point *i*, and $\overset{―}{d}$’ is the average across all the observed discrimination index in a session. *R*^*2*^ represents how well the model explains the data with values from 0 to 1, where *R*^*2*^ close to one indicates a better fitting of the model to the data.

## Results

We evaluated two traits of early stimulus processing while participantes developed perceptual learning and expertise: the rate of information encoding after stimulus onset and the onset of availability of stimulus information for encoding. To develop perceptual learning and low levels of perceptual expertise, participants completed six discrimination training sessions (Fig 1A) with a novel multi-exemplar category of Kanji stimuli with no meaning for Spanish-speaking participants. To assess the acquisition of perceptual expertise, we estimated the transfer of learning to novel Kanji stimuli as an index of the generalization of learning to the stimuli category. Finally, to examine early processing, we evaluated the effect of encoding durations at different levels of discrimination training. First, we show discrimination learning during training. Second, we show discrimination performance with increasing encoding durations at four levels of training. Third, we describe the transfer of discrimination learning to novel stimuli. Lastly, we show the estimation of the rate of information extraction after stimulus onset and the onset stimulus information available for encoding obtained by fitting the sensitivity data from the evaluation sessions.

### Perceptual learning during discrimination training

**Fig 2:**
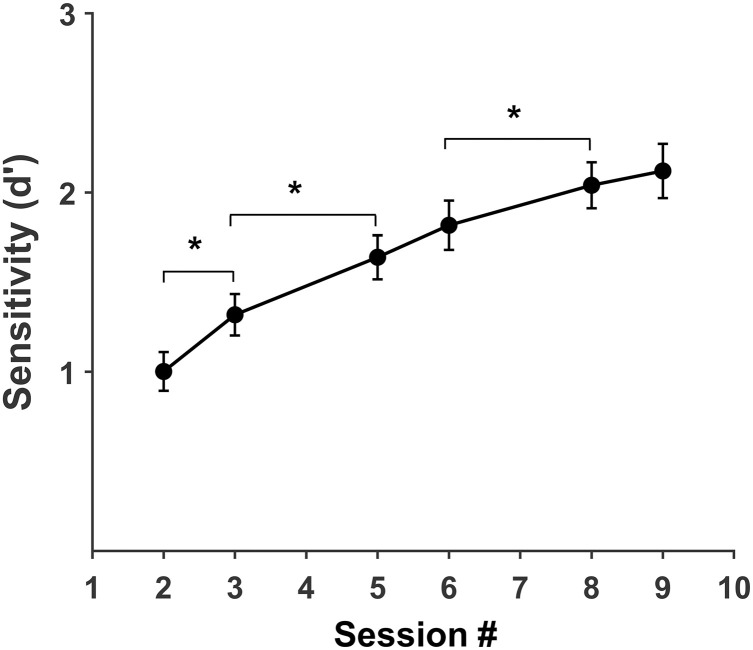
Sensitivity (*d’*) as a function of discrimination training. Mean *d’* values at each training session. Error bars represent the standard error of the mean. Asterisks indicate statistical differences between the successive sessions (*, paired t-test with BH correction).

Discrimination training with Kanji stimuli throughout six sessions gradually increased in performance (Fig 2). The mean sensitivity (*d’*) increases from *M* = 1.03, *SD* = 0.55 in the first session to *M* = 2.13, *SD* = 0.85 in the ninth session. One-factor repeated measures (session number) ANOVA conducted to evaluate the effect of training on performance shows a significant effect of training (*F* (2.95, 76.72) = 62.5, *p* < .001, η^2^_p_ = .706), corroborating that training improves discrimination of Kanji stimuli. T-tests for sensitivity at successive sessions show a significant increase from the second to the eighth session (*p* < .05, paired t-tests) with medium to high effect sizes (Cohen’s *d*: −.79, −1.27, −1.06, for session pairs 2–3, 3–5, 6–8, respectively), but no difference between the fifth and sixth sessions (*p* = .05) and the eighth and ninth sessions (*p* = .32). These results demonstrate that training gradually increases stimulus discriminability, in agreement with the development of perceptual learning and expertise.

### Training increases discrimination sensitivity and reduces encoding duration

**Fig 3:**
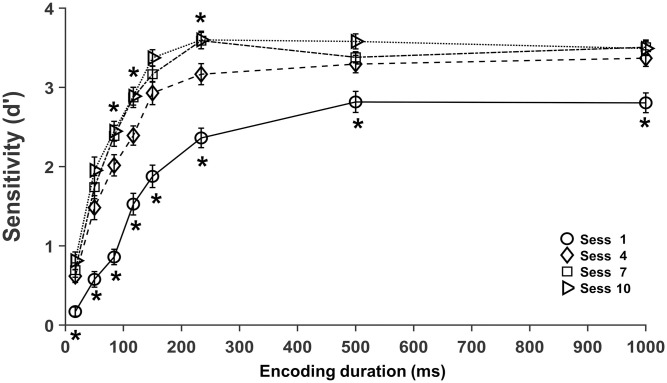
Sensitivity (d’) as a function of encoding duration at four levels of discrimination training. Mean *d’* in the first (circle), fourth (diamond), seventh (square) and tenth (triangle) sessions. Error bars represent the standard error of the mean. Asterisks indicate significant differences between sessions 1 and 4 (below session 1) and session 4 and 7 (over session 7) at each encoding duration (*, paired t-tests with BH correction).

The effect of discrimination training on early stimulus processing was assessed as the sensitivity at four levels of training: no training (session1), low training (session4), medium training (session7) and high training (session10) with encoding durations from 17 to 1000 ms (Fig 3).

Based on the gradual increase in sensitivity across training sessions (Fig 2), we anticipated an increase in the sensitivity between no training and low training, low and medium training, and a minor effect between medium and high training. We also anticipated increased sensitivity with longer stimuli at all training levels and the saturation of sensitivity with shorter stimuli at increasing levels of training [17]. Our results show increases in sensitivity with more training at all encoding durations, with a larger effect between the no training and low training, a smaller increase between low and medium training, and no further change between medium and high training (Fig 3). Performance for each encoding time and training level are shown in Table 1. A 4 (session) * 8 (encoding duration) repeated measures ANOVA was conducted to evaluate the effect of training and encoding time on performance (*d’*). We found main effects of training (*F*(1.59, 41.43) = 62.78, **p* *< .001, η^2^_p_ = .707) and encoding duration (*F*(3.37, 87.61) = 293.58, **p* *< .001, η^2^_p_ = .919), indicating that more training and longer encoding times improve discrimination. Moreover, there was a significant interaction between training and encoding duration (*F*(10.4, 270.67) = 4.13, **p* *< .001, η^2^_p_ = .137), indicating that the effect of training on sensitivity vary with encoding time. Specifically, training has a greater effect on sensitivity at intermediate durations.

**Table 1:** Sensitivity (d’) values as a function of encoding duration and discrimination training.

Encoding duration (ms)	Session1	Session 4	Session 7	Session 10
17	0.169 ± 0.303	0.618 ± 0.417	0.694 ± 0.471	0.813 ± 0.576
50	0.581 ± 0.511	1.485 ± 0.783	1.741 ± 0.762	1.953 ± 0.874
84	0.860 ± 0.501	2.015 ± 0.698	2.388 ± 0.685	2.446 ± 0.648
117	1.529 ± 0.709	2.406 ± 0.640	2.873 ± 0.666	2.889 ± 0.584
250	1.884 ± 0.737	2.936 ± 0.756	3.166 ± 0.518	3.373 ± 0.529
234	2.364 ± 0.634	3.187 ± 0.715	3.589 ± 0.535	3.599 ± 0.574
500	2.820 ± 0.685	3.293 ± 0.569	3.379 ± 0.364	3.579 ± 0.494
1000	2.805 ± 0.647	3.368 ± 0.543	3.520 ± 0.472	3.491 ± 0.467

Mean ± SD of sensitivity with encoding durations at each evaluation session.

Differences in sensitivity between sequential evaluation sessions were assessed by paired t-tests. Significant increases were obtained from no training to low training (sessions 1 and 4, *t* (26) ≤ −2.804, **p* *< .01, medium to large effect sizes Cohen’s *d* = − 0.54 to – 2.06) at all encoding times (Fig 3), from low to medium training (sessions 4 and 7, *t* (26) ≤ −2.257**, p* *< .05, medium *t*o large effect sizes Cohen’s *d* = − 0.43 to – 0.77) at intermediate encoding durations (84, 117 and 234 ms) except for 150 ms, although additional training (session 10) increases performance at 150 ms, and no difference between medium and large training (sessions 7 and 10) with all encoding durations, except with 500 ms (*t* (26) ≤ −2.494**, p* *= .02, medium effect size Cohen’s *d* = − 0.48), where performance wi*t*h medium training did not increase relative to low training (Fig 3). These results demonstrate that two training sessions increase performance with all encoding durations, four training sessions improve sensitivity only for intermediate encoding times (84, 117, 234 ms) except with 150 ms, revealing a saturation of the outcome of training on discrimination performance with brief stimuli (17 and 50 ms) and long stimuli (500 and 1000 ms). Finally, additional training did not increase sensitivity with the majority of the encoding durations, indicating saturation or slowing down of the impact of training on performance.

**Fig 4:**
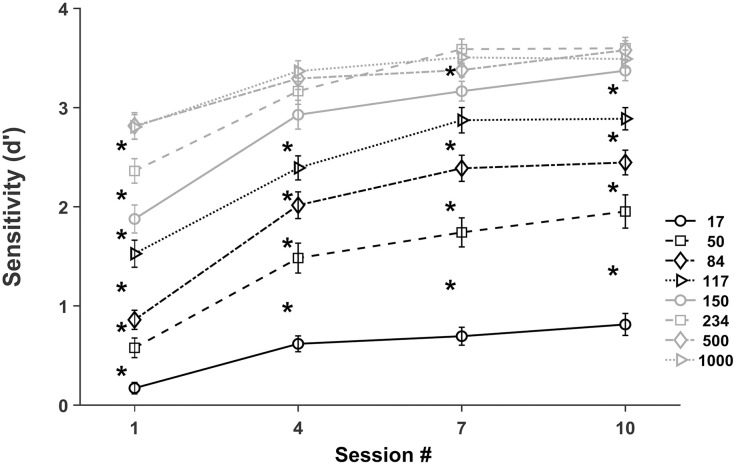
Sensitivity (d’) as a function of encoding duration at four levels of discrimination training. Mean *d’* values for encoding durations (ms) 17, 50, 84, 117 (black) and 150, 234, 500 and 1000 (grey) with no training and low, medium and high training (sessions 1, 4 7 and 10). Error bars represent the standard error of the mean. Asterisks indicate significant differences in sensitivity (*,paired t-test with BH correction) for successive encoding durations (17 to 50, 50 to 84, 84 to 117, 117 to 150, 150 to 224 and 234 to 500).

Statistical differences in performance between growing encoding durations (Fig 4) show that sensitivity increased progressively from 17 to 500 ms with no training (*t* (26) ≤ −3.084, **p* *< .01, medium to large effect sizes Cohen’s *d* = − 0.59 to – 1.25); from 17 to 150 ms with low training (*t* (26) ≤ −2.978, **p* *< .01, medium *t*o large effect sizes Cohen’s *d* = − 0.57 to – 1.55); from 17 to 234 ms with medium training (*t* (26) ≤ −2.177, **p* *< .01, medium to large effec*t* sizes Cohen’s *d* = − 0.42 to – 1.50), except from 117 to 150 ms; and from 17 to 150 ms with large training (*t* (26) ≤ −4.136, **p* *< .01, medium to large effect sizes Cohen’s *d* = − 0.80 *t*o – 1.76). These results corroborate previous evidence showing increasing discrimination sensitivity with longer encoding times up to a plateau and a reduction of the encoding time required to reach the plateau performance with more training. Thus, maximum sensitivity is reached at 500 ms with no training (session 1), at 150 ms with low training (session 4), at 234 ms with intermediate training (session 7) and at 150 ms with high training (session 10). In summary, longer encoding times are required for discrimination of unfamiliar stimuli and shorter encoding times are sufficient to achieve maximum discrimination of familiar stimuli.

The onset of stimulus information extraction estimated from performance greater than chance (*d’* = 0) was assessed by a one sample t-test on performance at 17 ms and *d’* = 0, for each level of training. Performance was just above chance with 17 ms in with no training (t(26) = 2.95, p = 0.007, 95% confidence intervals.051 −.290, medium effect size Cohen (d) = 0.57), but above chance with low (t(26) = 7.70, p < 0.001, 95% confidence intervals.453 −.783, large effect size Cohen (d) = 1.48), medium (t(26) = 7.66, p < 0.001, 95% confidence intervals.508 −.881, large effect size Cohen (d) = 1.47 and high training (t(26) = 7.33, p < 0.001, 95% confidence intervals.585 - 1.041, large effect size Cohen (d) = 1.41). Taken together, these results suggest that discrimination training leads to an earlier onset of the availability of information for encoding, from about 17 ms without training to a shorter value with two or more training sessions.

### Training performance generalizes to novel Kanji exemplars

**Fig 5:**
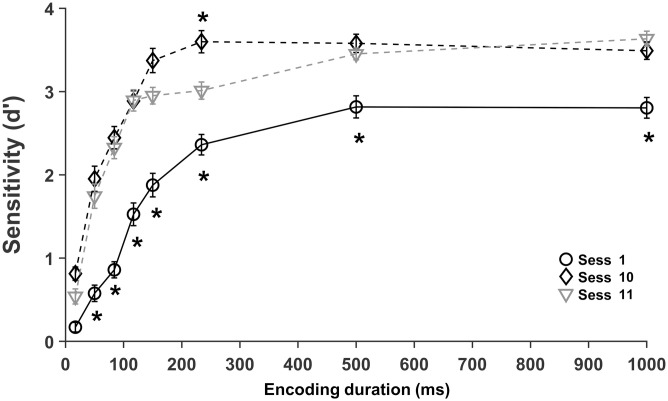
Sensitivity (d’) as a function of encoding duration for trained and novel Kanji stimuli in trained and novice participants. Mean *d’* values with novel Kanji stimuli in trained (session 11, triangle) and untrained participants (session 1, circle) compared with mean *d’* values with trained stimuli in trained participants (session 10, diamond). Error bars represent the standard error of the mean. Asterisks indicate significant differences between sessions 11 and 1 (below session 1) and session 11 and 10 (over session 11) for each encoding duration (*, paired t-tests with BH correction).

To assess the generalization of the discrimination learning to the Kanji category, participants performed the generalization session with novel Kanji exemplars. Sensitivity in trained participants with novel (session 11) and familiar (session 10) stimuli was mostly similar, but markedly larger than in untrained participants with unfamiliar stimuli (session 1) (Fig 5). Sensitivity values with encoding durations of 17, 50, 84, 117, 150, 234, 500 and 1000 ms were *M* = .54, *SD* = 0.58, *M* = 1.75, *SD* = 0.77, *M* = 2.35, *SD* = 1.05, *M* = 2.90, *SD* = 0.72, *M* = 2.94, *SD* = 0.65, *M* = 3.04, *SD* = 0.53, *M* = 3.45, *SD* = 0.44 and *M* = 3.66, *SD* = 0.55, respectively. Paired t-test for sensitivity with trained Kanji (session 10) was similar to that with novel stimuli (session 11) for the majority of encoding times (17, 50, 84, 117, 150 500 and 1000 ms, *t* (15) ≤ 2.655, **p* *≥ .05) but lower for 234 ms (*t* (15) ≥ 3.313, **p* *= .005, medium effect size Cohen’s *d* = 0.83). In contrast, the majority of sensitivity values with novel stimuli in trained participants (session 11) were substantially greater than those in untrained participants with novel stimuli in the first session (*t* (15) ≤ −2.918, **p* *≤ .02, medium to large effect sizes (Cohen’s *d* = − 0.73 to – 1.82), except for 17 ms (*t* (15) = − 2.114, **p* *= .05, medium effect size Cohen’s *d* = 0.53). Taken together, these results show that discrimination training generalizes to novel exemplars of the Kanji category, indicating the acquisition or processing abilities for the detection of relevant stimulus features or patterns for discrimination, characteristic of perceptual learning and expertise.

### Discrimination training leads to increases the rate of performance change with encoding times and earlier onset stimulus encoding

The impact of discrimination training in the rate of performance change with increasing encoding times and the onset of availability of stimulus information for encoding was evaluated by fitting an exponential function to the sensitivity data (see methods).

**Fig 6:**
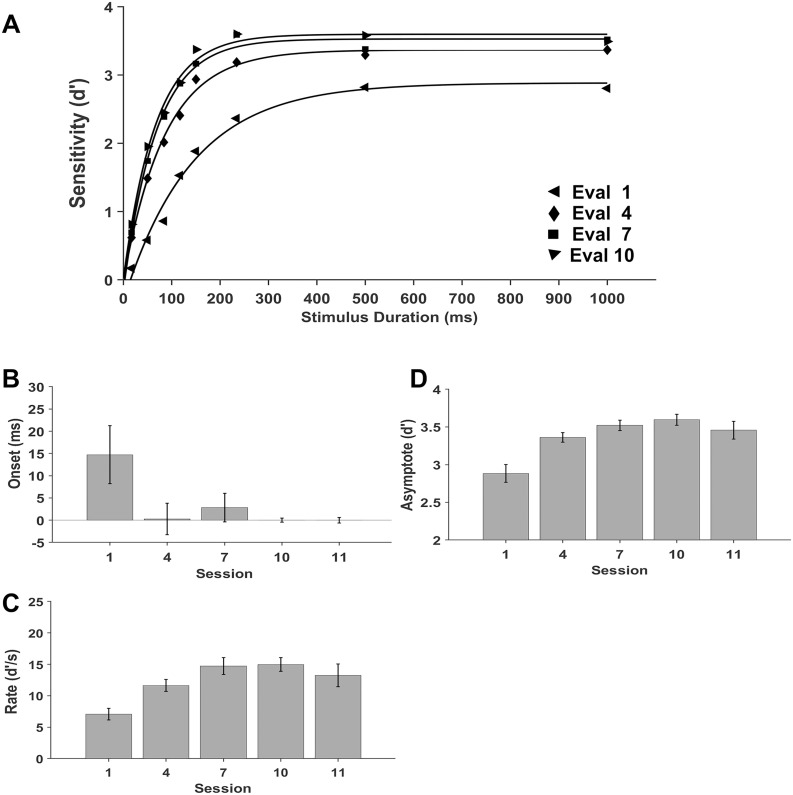
Model fit to mean sensitivity data as a function of encoding duration at each level of discrimination training: fitting and parameters. A. Discrimination performance (d’) with Kanji stimuli with no training and low, medium and high training in sessions 1, 4, 7 and 10, respectively, was fitted by a shifted exponential function. B. Extrapolated mean encoding duration for performance at chance at each session. C. Mean values of the initial rate of stimulus encoding at each session. D. Mean values of the asymptote at each session. Error bars represent the standard error of the mean.

First, we estimated the encoding duration where the sensitivity curve intersects the x axis, representing chance-level performance. The fit of the model to the sensitivity data with no training (*R*^2^ = .984), with low training (*R*^2^ = .993), with medium training (*R*^2^ = .991), with high training (*R*^2^ = .989) and in the generalization (*R*^2^ = .975) session (Figs 6A) yielded an estimated encoding time for performance at chance level of 15 ms with no training. Performance at chance level decreased to near 0 ms with two of more training sessions (Fig 6 B and S6 Fig). The estimated encoding duration for performance at chance level in ms are *M* = 14.71, *SE *= 6.52 with no training, *M* = 0.28, *SE* = 3.56 with low, *M* = 2.83, *SE* = 3.22 with medium and *M* = 0.00, *SE* = 0.46 with high training. In addition, the performance at chance level was *M* = 0.00, *SD* = 0.63 for novel Kanji stimuli with high training. These results suggest a reduction of the onset of stimulus encoding by approximately 15 ms with two or more training sessions with trained and novel stimuli.

Second, the rate of change in performance with encoding times was estimated as the slope of discrimination performance with increasing encoding times. This slope increases gradually from the first to the seventh sessions (Fig 6 C and S1-S6 Fig), with values expressed as d’/s of *M* = 7.08, *SE* = .93, *M* = 11.62, *SE* = .95, *M* = 14.74, *SE* = 1.35, *M* = 14.96 and *SE* = 1.09 with no training, low, medium and high levels of training, respectively. The slope in the generalization session with novel Kanji stimuli is and *M* = 13.25, *SE* = 1.80. These results suggest that discrimination training speeds up the information extraction after stimulus onset, which parallels the development of perceptual learning and expertise in the training sessions. In sum, discrimination training modifies early stimulus processing by increasing the speed of information extraction after stimulus onset and inducing an earlier onset of the information extraction.

Finally, the maximum performance or asymptote gradually increases with training (Fig 6 D). The asymptote expressed as d’ are *M* = 2.88, *SE* = .12, *M* = 3.36, *SE* = .06, *M* = 3.53, *SE* = .07 and *M* = 3.60, *SE* = .07 with no training, low, medium and high levels of training, respectively. With novel Kanji exemplars in the generalization session, the asymptote was similar to that with trained stimuli (*M* = 3.47, *SE* = .12). These results demonstrate that discrimination training increases the maximum sensitivity, in agreement with the development of perceptual learning and expertise.

## Discussion

We used a novel approach to evaluate early stimulus processing during perceptual learning and expertise. Here, we assessed the speed of information encoding after the stimulus and the onset of availability of stimulus information for encoding. We demonstrate that perceptual learning through discrimination training leads to a gradual increase in the rate of performance change with encoding duration, revealing faster stimulus information extraction. In addition, we corroborate the earlier availability of stimulus information for encoding [17] after two training sessions, indicating an earlier onset of information extraction.

First, an essential issue for the interpretation of our results is that discrimination training lead to perceptual learning and low levels of perceptual expertise supported by the generalization session. The progressive increase in sensitivity during discrimination training (Fig 2) and the generalization of learning to novel Kanji exemplars support the development of perceptual learning and expertise. Increases in sensitivity across training sessions exhibit medium to large effect sizes, except between sessions 5 and 6 and 8 and 9 (Fig 1), in agreement with lab trained and natural experts in individuation [13], recognition [14,15] and categorization [24,44] tasks. Moreover, our results match those obtained in discrimination training with stimuli of medium complexity [22].

Several features of the training task were relevant in the development of perceptual learning and expertise. Similar training tasks were previously used in object discrimination [13,22,43] to develop object learning and perceptual expertise. Early studies on perceptual expertise implemented tasks such as individuation with naming or verification of stimuli [15,24], but naming or labeling is not required for perceptual expertise [51]. Moreover, an encoding duration of 100 ms is sufficient for the detection and individuation of complex stimuli in experts [17,44], the acquisition of perceptual expert performance [11,42] as well as the decoding of images in the cortex [52]. Stimulus encoding durations were defined by backward masking [17], and retinal matching of the stimuli was circumvented by the rotation of the second stimuli [11,42], previously described in perceptual learning of faces [49] and repetition priming for common objects [50].

Besides, the generalization session demonstrates the overall transfer of learning to the Kanji category (session 11, Fig 5), in agreement with the acquisition of perceptual expertise [1,43]. Although the generalization session was completed by a subset of participants, because some participants were unable complete the generalization session, our results reveal a full transfer of learning to novel stimuli for the majority of stimulus durations. This is in contrast with the difference in performance between trained and untrained participants with novel stimuli for most stimulus durations supported by medium to large effect sizes (sessions 1 and 11).

In addition to the development of perceptual learning and expertise, stimulus exposure promotes familiarity [42,53] and repetition priming [53–56]. Familiarity, a type of recognition memory, speeds up the detection and categorization of brief stimuli at the basic level [44] and enhances the amount of coarse information available in the first milliseconds after stimulus onset [57]. Because familiarity relies on previous exposure, in our work one or two training sessions are sufficient to develop stimuli familiarity [42]. However, we found that discrimination increased progressively with training, indicating that familiarity does not account for the sensitivity improvements during training, although it may contribute to performance in specific sessions. Besides, similar performance with familiar (trained) and unfamiliar (novel) stimuli (sessions 10 and 11, respectively), is inconsistent with familiarity underlying sensitivity improvement during training.

Regarding repetition priming, an implicit memory that results in increased performance and faster responses with familiar and unfamiliar stimuli [55,58–60], that develops after a single or a few exposures to the stimuli in a single session [61] is unlikely the main factor underlying performance improvement in training sessions.Finally, the training task likely involves procedural learning such as decisional and motor readiness [62]. We cannot rule out that a fraction of the sensitivity improvement is based on learning procedural aspects of the task, probably achieved with low levels of training. In summary, sensitivity improvements during discrimination training and generalization of this learning to novel stimuli support the developed perceptual learning and low levels of perceptual expertise [1,11,43,63].

Secondly, Kanji discrimination with brief encoding times at four levels of training show that both, more training and longer encoding times increase sensitivity (Figs 3 and 4), in agreement with prior findings in discrimination [17], detection [21] and identification [14] tasks. Interestingly, the effect of training on performance varies with encoding duration (Figs 3). Indeed, low levels of training (two sessions) lead to better discrimination with all encoding durations (from 17 to 1000 ms), medium levels of training (four sessions) further boost performance for intermediate encoding times (80–234 ms) with the exception of 150 ms, with medium to large effect sizes, revealing a saturation of the effect of training with brief (17 and 50 ms) and long (≥ 500 ms) stimuli. Interestingly, there was a significant increase in performance at 150 ms with high levels of training (Fig 3). Finally, two additional training sessions did not significantly increase performance for all encoding times, indicating the saturation or slowing down of the effect of training in stimulus processing. These results show a reduction of the stimulus processing time with increasing training levels. Previous evidence showed a reduction in the processing time required for texture detection with increasing amounts of training [21] and a similar difference in discrimination sensitivity between face and car experts, relative to novices, at various encoding durations [17].

Furthermore, increasing encoding times gradually improve performance up to a maximum or plateau (Fig 4) with medium to large effect sizes for all training levels, in agreement with greater stimulus information extraction and encoding as stimulus durations increase. Training reduces the encoding duration required for maximum performance from 500 ms without training to 150 ms after two and six training sessions and 234 ms after four training sessions. Interestingly, a duration of 180 ms was sufficient for maximum stimulus encoding in the identification of familiar objects [44]. In our data a *d prime* = 1.68 (80% correct responses) was achieved with encoding durations of 140, 60 and 45 ms with no training and two and four training sessions, respectively (Figs 4 and S6), demonstrating a reduction of encoding duration required to achieve the same performance. Likewise, training of a texture detection task reduced stimulus processing duration from 120 to 40 ms [21]. In summary, discrimination training reduces the encoding time required for a given performance, suggesting a faster speed of stimulus processing in the first milliseconds after stimulus onset or, alternatively, a reduction in the amount of information needed for encoding as in visual repetition priming [60]. Thus, the development of perceptual learning and expertise reduce the encoding time required to achieve a given performance.

Third, our results show that perceptual learning and expertise modulate early stimulus processing by an increase in performance rate with encoding time as well as an earlier onset of stimulus information availability for encoding (Figs 6 and S6), obtained by fitting the exponential model to the data. The increase in the rate of performance is consistent with a reduction of the stimulus processing time over seven sessions in a texture detection task [21], but contrasts with previous evidence on car and face discrimination in experts and novices, which found no changes in the rate of performance [17]. These conflicting results might arise from differences in the number of trials, which in the study of Curby and Gauthier (Figs 1A and 4 A [17]) have significant errors, which could conceal differences in rates. In addition, in our study participants had substantially more task practice across six discrimination training sessions. Interestingly, the sensitivity values in the evaluation sessions are comparable to those of experts and novices in Curby and Gauthier [17], indicating a similar discrimination performance [17]. Further studies should be done to evaluate the effect of stimuli repetition and extent of task practice on discrimination.

The earlier availability of stimulus information for encoding, estimated from the fitting and from the performance greater than chance with increasing levels of discrimination training, agree qualitatively with the earlier onset found in experts relative to novices [17]. Yet, our onset values are much lower than the ones observed with inverted (60 ms) and upright faces (27 ms) and in car novices (55 ms) and experts (12 ms) [17]. Interestingly, evidence from MEG/fMRI signals with familiar and unfamiliar images showed an earlier availability of stimulus information for encoding in familiar relative to unfamiliar stimuli [45,52,57] which might explain in part this discrepancy in the values. On the other hand, 20 ms durations were sufficient for the categorization of complex images [64] and for a performance greater than chance in texture detection [21]. Nonetheless, the onset times obtained from the fitting are an extrapolation, as the minimum encoding time in our study was 17 ms. In consequence; the onset values represent a lower limit for the onset of the availability of stimulus information. Most likely, a minimum encoding duration must be achieved to obtain a performance better than chance. Additional studies with encoding durations between 1 and 50 ms should be done to determine the onset of the availability of stimulus information and its modulation by discrimination training. Finally, maximum performance or asymptote shows a progressive enhancement with increasing amounts of training, in agreement with previous studies on discrimination training [22] and discrimination in experts compared to novices [17].

The exact strategy for discrimination of Kanji stimuli underlying perceptual learning and expertise in our experimental design was not identified. Yet, previous studies suggest that discrimination of complex stimuli is based on the extraction of the relevant features necessary for discrimination of a specific stimuli category, which may include pattern discrimination [65] and holistic processing [41] and with extended practice the relevant features are extracted with greater selectivity and fluency [20]. Previous studies showed that discrimination training with complex stimuli increased the strength of the neural responses and its distribution in object-specific visual areas to trained stimuli [22], supporting the development of perceptual expertise.

Different mechanisms have been suggested for perceptual learning and perceptual expertise acquisition [12,18,20,34,40,66–68]. Improvements in performance have been attributed to an enhanced differentiation of the stimuli relying on a finer tuning of neurons in primary and higher cortices for simple features [35,36,66] and object-selective cortices for complex stimuli [22]. Besides, faster responses or perceptual fluency could rely on a switch from later to earlier cortices during perceptual learning [66] or on automation of stimuli processing [69]. Our results are consistent with greater and faster differentiation of the stimuli. Alternative mechanisms for better performance with stimuli familiarity and expertise include a higher resolution of short-term memory (STM) representations [70] and greater encoding and consolidation of familiar stimuli in STM [71,72]. Although we cannot draw conclusions on the mechanisms underlying performance improvement with training, our results are consistent with higher resolution of neural representations and an earlier availability of stimulus information for discrimination. Further studies are necessary to distinguish the effects of discrimination training on perception and STM processes.

While the results presented here provide additional evidence that discrimination training and the acquisition of low levels of expertise modulate stimulus processing in the first milliseconds after stimulus onset, they also have several limitations. First, perceptual expertise in discrimination and identification develops slowly with increasing levels of training [9,14,15,24] and our measurements included a limited number of training sessions because the difficulty in maintaining participants coming to the lab for several consecutive sessions. In consequence, the results presented here do not represent natural experts but more likely low levels of perceptual expertise. Second, the estimation of the onset of the availability of stimulus information is based on an extrapolation of the data and does not represent a direct measurement of the encoding duration at which performance raises above chance. Third, although the estimated rates of performance change with encoding duration suggests greater rates with more training; further studies are needed to confirm these results. Fourth, a fraction of performance improvement in the initial session might include learning of procedural aspects of the task.

In sum, we provide evidence for the modulation of early stimulus processing while participants developed perceptual learning and low levels of expertise. We show a gradual increase in sensitivity with both, more training and longer encoding durations. Moreover, the effect of training on sensitivity is greatest with intermediate encoding durations. Modeling of the sensitivity data as a function of training and encoding times revealed a faster rate of performance increase with encoding duration and an earlier availability of stimulus information for encoding. The effect of training on the rate of performance parallels the sensitivity change with training, suggesting its association with the development of perceptual learning and expertise. In contrast, the earlier availability of stimulus information for encoding is achieved with low levels of training, suggesting an association with the acquisition of familiarity with the stimuli. This work provides additional evidence on the impact of discrimination training on stimulus processing in the first milliseconds after stimulus onset. The findings presented here suggest that perceptual learning and expertise for a complex stimulus category speeds up stimulus information extraction in the first milliseconds after stimulus onset and result in an earlier onset stimulus information extraction for encoding.

## Supporting information

S1 Fig Performance (d’) during Kanji discrimination in evaluation session 1. The shifted exponential function fitted to the means sensitivity data resulted in a rate of approach to asymptote of 7.08, and performance onset (intercept) of 15 ms and asymptote of 2.88 (r^2^ = .9844).
(TIF)

S2 Fig Performance (d’) for Kanji discrimination in evaluation session 4. The shifted exponential function fitted to the means sensitivity data resulted in a rate of approach to asymptote of 11.6, and performance onset (intercept) of.3 ms and asymptote of 3.37 (r^2^ = .9931).
(TIF)

S3 Fig Performance (d’) for Kanji discrimination in evaluation session 7. The shifted exponential function fitted to the means sensitivity data resulted in a rate of approach to asymptote of 14.7, and performance onset (intercept) of 2.8 ms and asymptote of 3.53 (r^2^ = .9912).
(TIF)

S4 Fig Performance (d’) for kanji discrimination in evaluation session 10. The shifted exponential function fitted to the means sensitivity data resulted in a rate of approach to asymptote of 15.0, and performance onset (intercept) of.0 ms and asymptote of 3.59 (r^2^ = .9890).
(TIF)

S5 Fig Performance (d’) for Kanji discrimination in evaluation session 11. The shifted exponential function fitted to the means sensitivity data resulted in a rate of approach to asymptote of 13.3, and performance onset (intercept) of.0 ms and asymptote of 3.473 (r^2^ = .9757).
(TIF)

S6 Fig Performance (d’) during Kanji discrimination in the first 100 ms after stimulus onset for evaluation sessions. The shifted exponential function fitted to the means sensitivity data for evaluation sessions 1 (+). 4 (■), 7 (●) and 10 (*), corresponding to no training and low, medium and high training, respectively. The intercept of the curve with the 0 in the y axis (d’) correspond to the estimated onset of stimulus information extraction.
(TIF)
