# Supplementary figures and images for: Visual discrimination training increases the speed stimulus processing and leads to an earlier onset of stimulus encoding
Source: PLoS One. 2025 Aug 18;20(8):e0330284. doi: 10.1371/journal.pone.0330284 (PMC12360524; doi:10.1371/journal.pone.0330284)

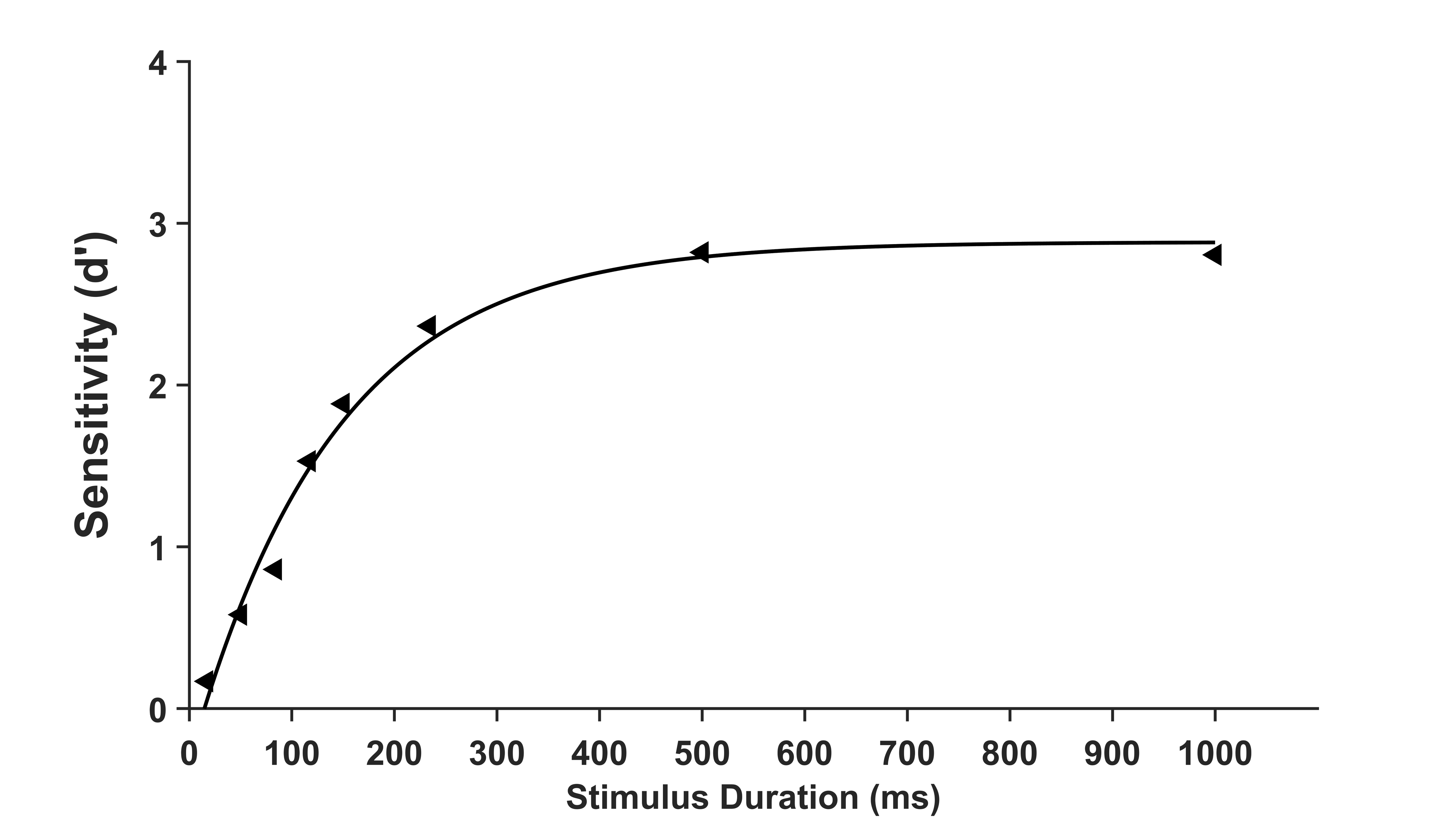

Supplement: S1 Fig — The shifted exponential function fitted to the means sensitivity data resulted in a rate of approach to asymptote of 7.08, and performance onset (intercept) of 15 ms and asymptote of 2.88 (r2 = .9844). (TIF) [file pone.0330284.s001.tif]

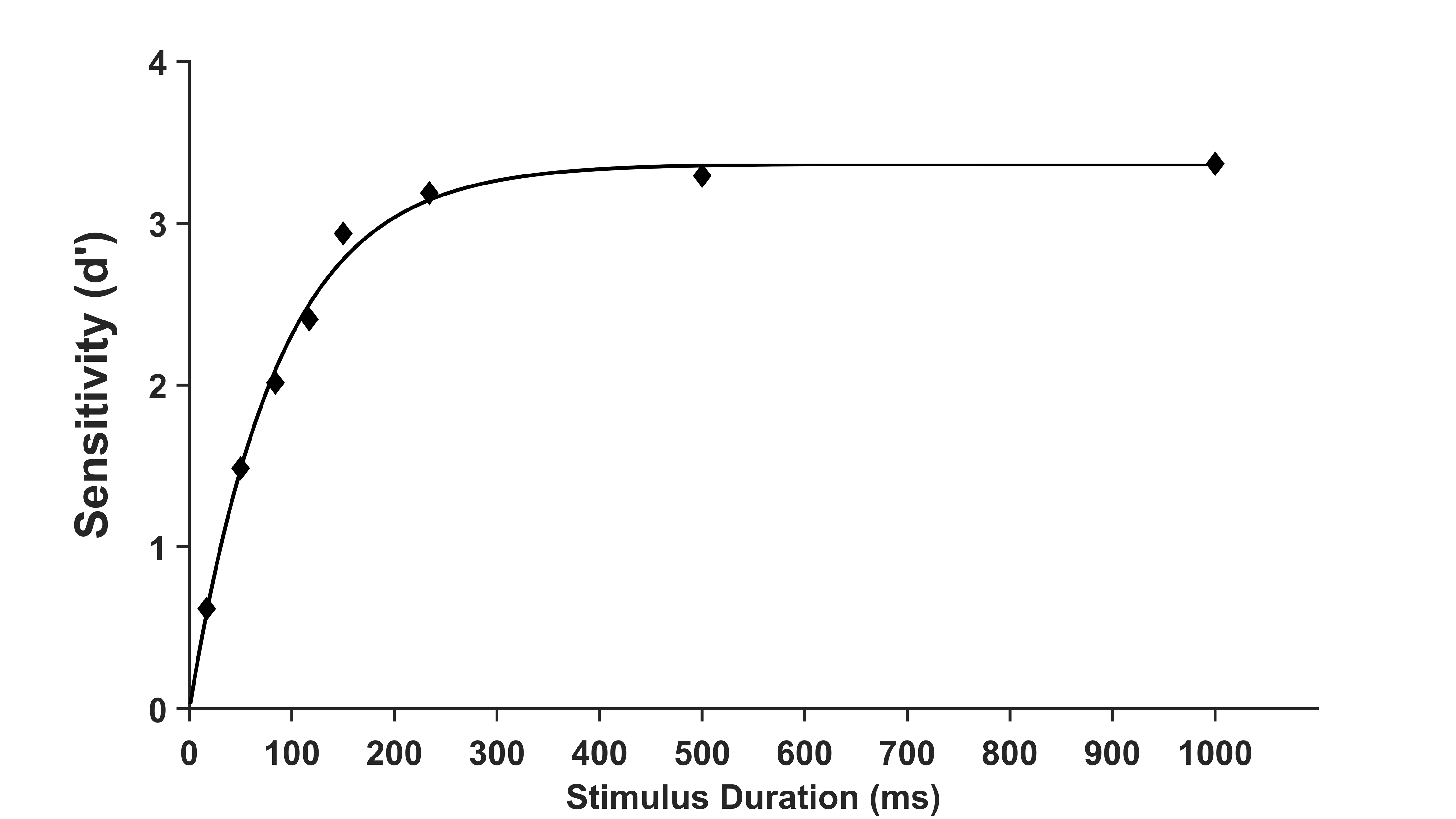

Supplement: S2 Fig — The shifted exponential function fitted to the means sensitivity data resulted in a rate of approach to asymptote of 11.6, and performance onset (intercept) of.3 ms and asymptote of 3.37 (r2 = .9931). (TIF) [file pone.0330284.s002.tif]

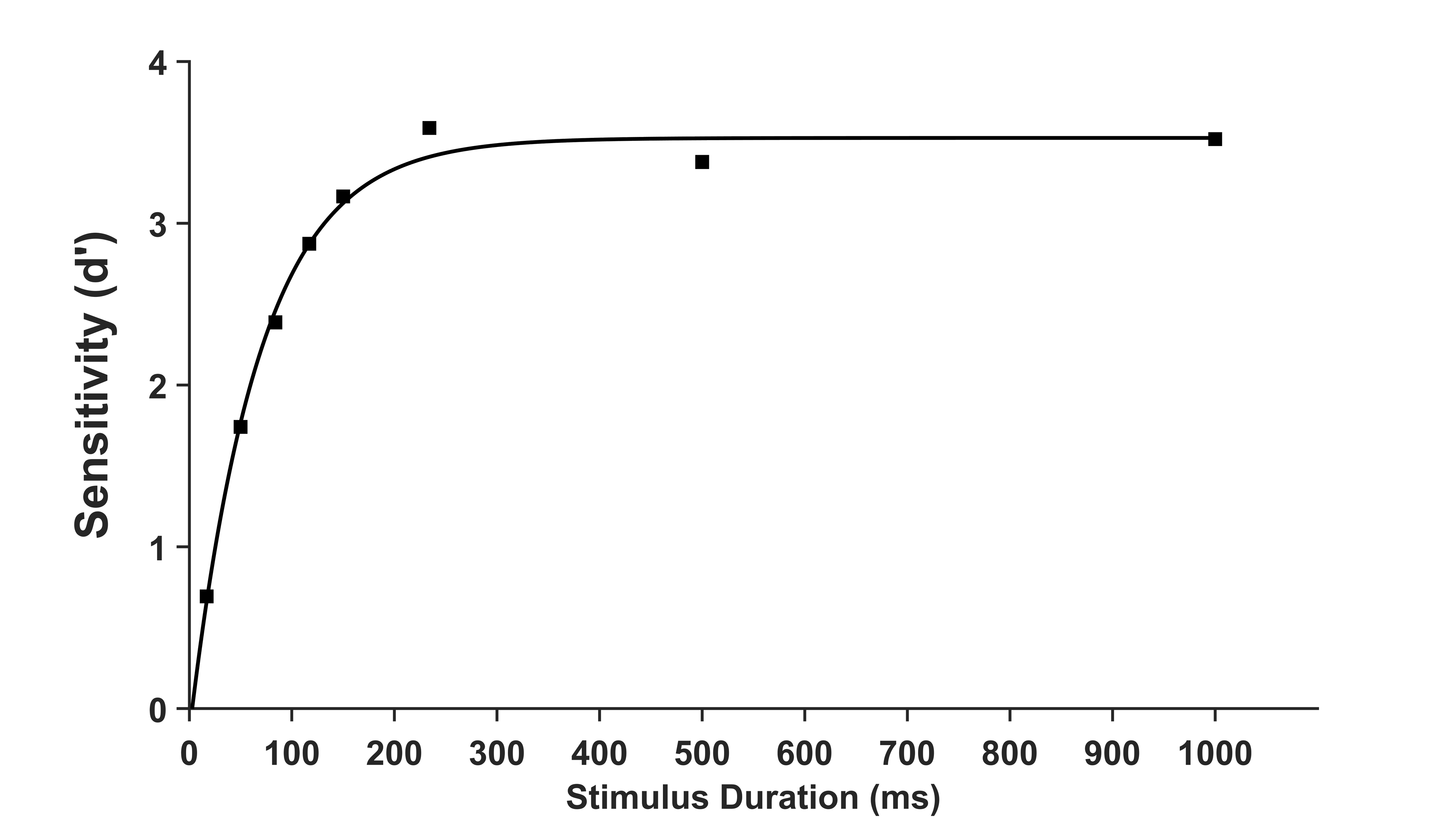

Supplement: S3 Fig — The shifted exponential function fitted to the means sensitivity data resulted in a rate of approach to asymptote of 14.7, and performance onset (intercept) of 2.8 ms and asymptote of 3.53 (r2 = .9912). (TIF) [file pone.0330284.s003.tif]

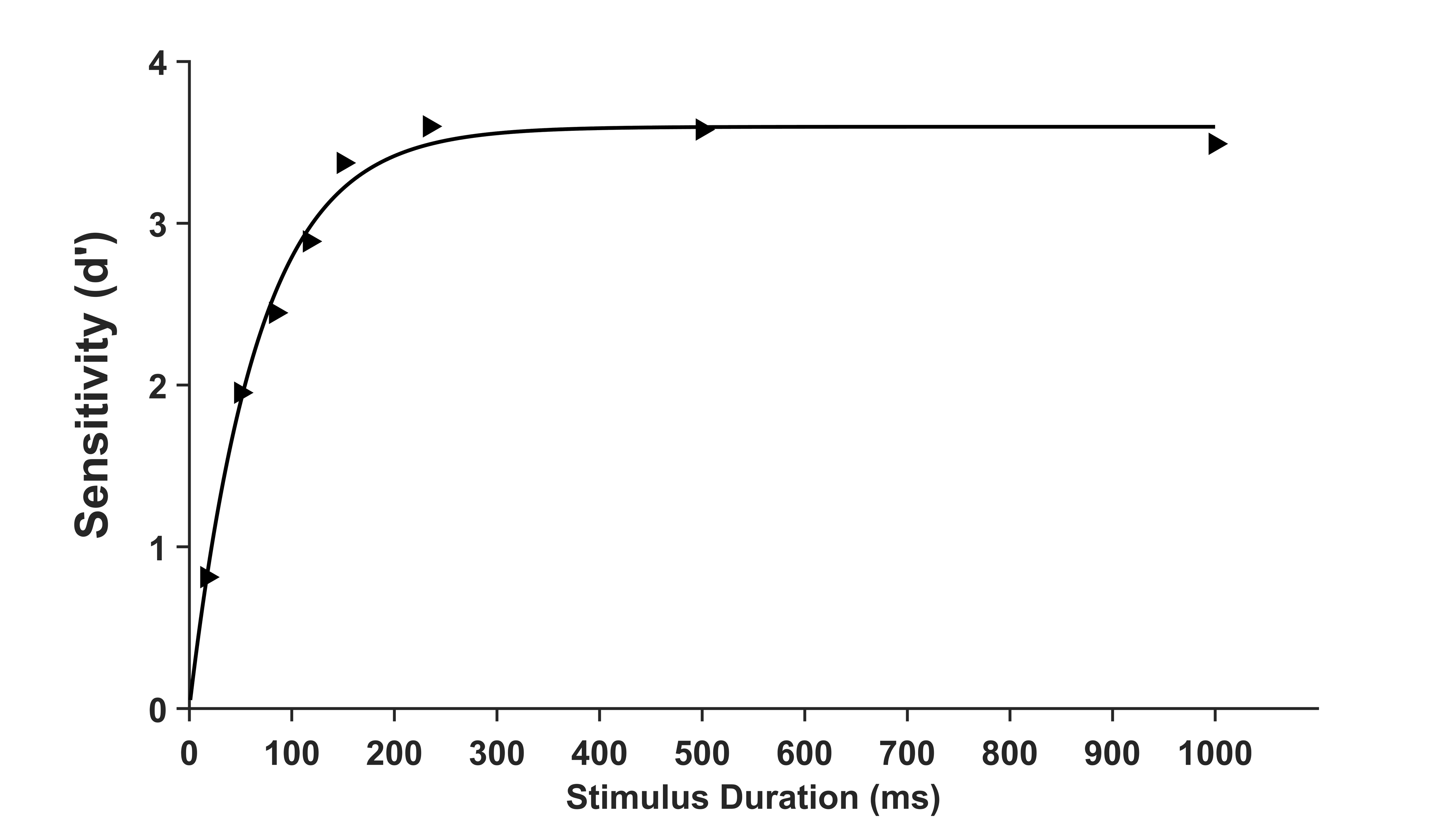

Supplement: S4 Fig — The shifted exponential function fitted to the means sensitivity data resulted in a rate of approach to asymptote of 15.0, and performance onset (intercept) of.0 ms and asymptote of 3.59 (r2 = .9890). (TIF) [file pone.0330284.s004.tif]

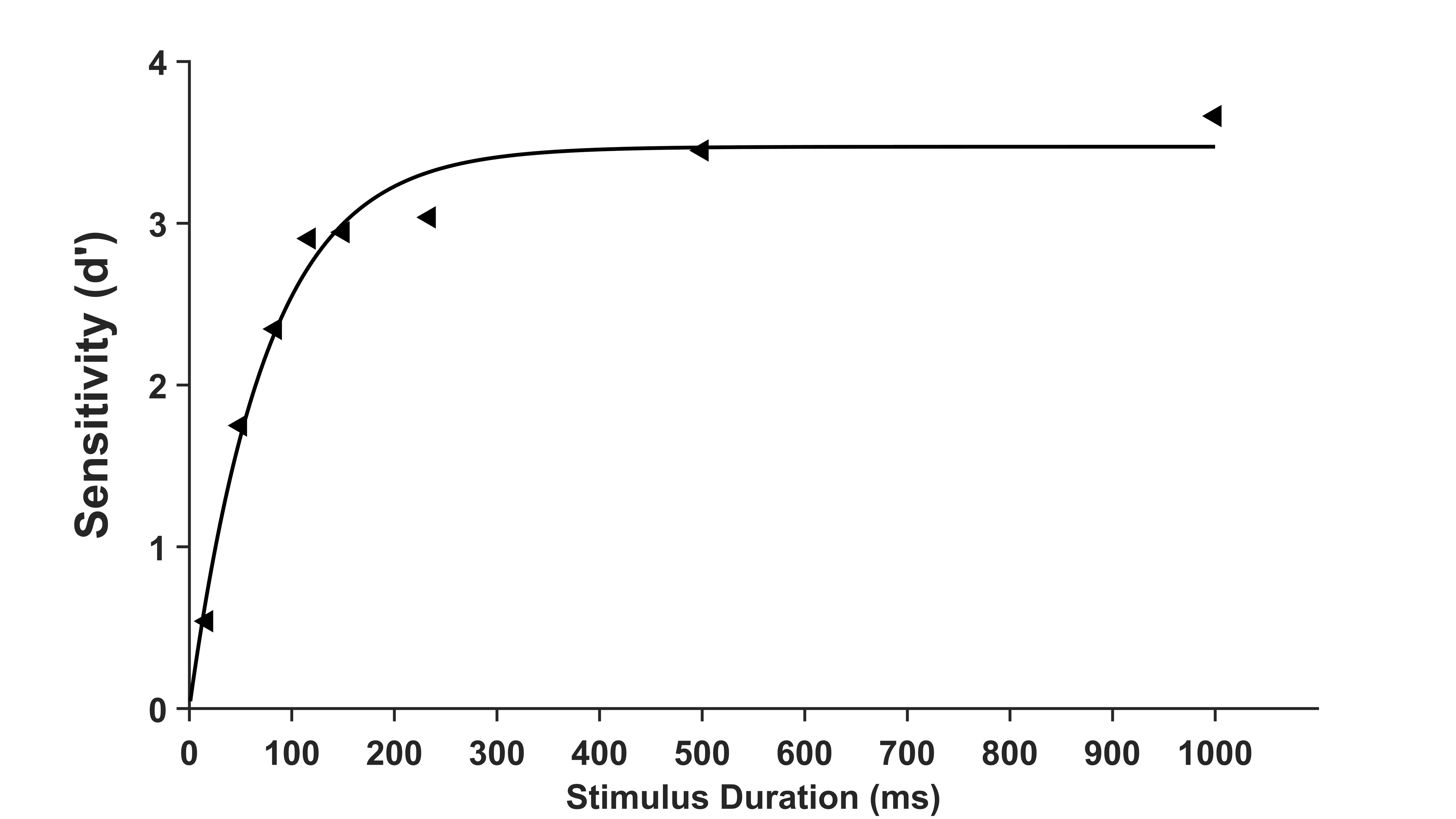

Supplement: S5 Fig — The shifted exponential function fitted to the means sensitivity data resulted in a rate of approach to asymptote of 13.3, and performance onset (intercept) of.0 ms and asymptote of 3.473 (r2 = .9757). (TIF) [file pone.0330284.s005.tif]

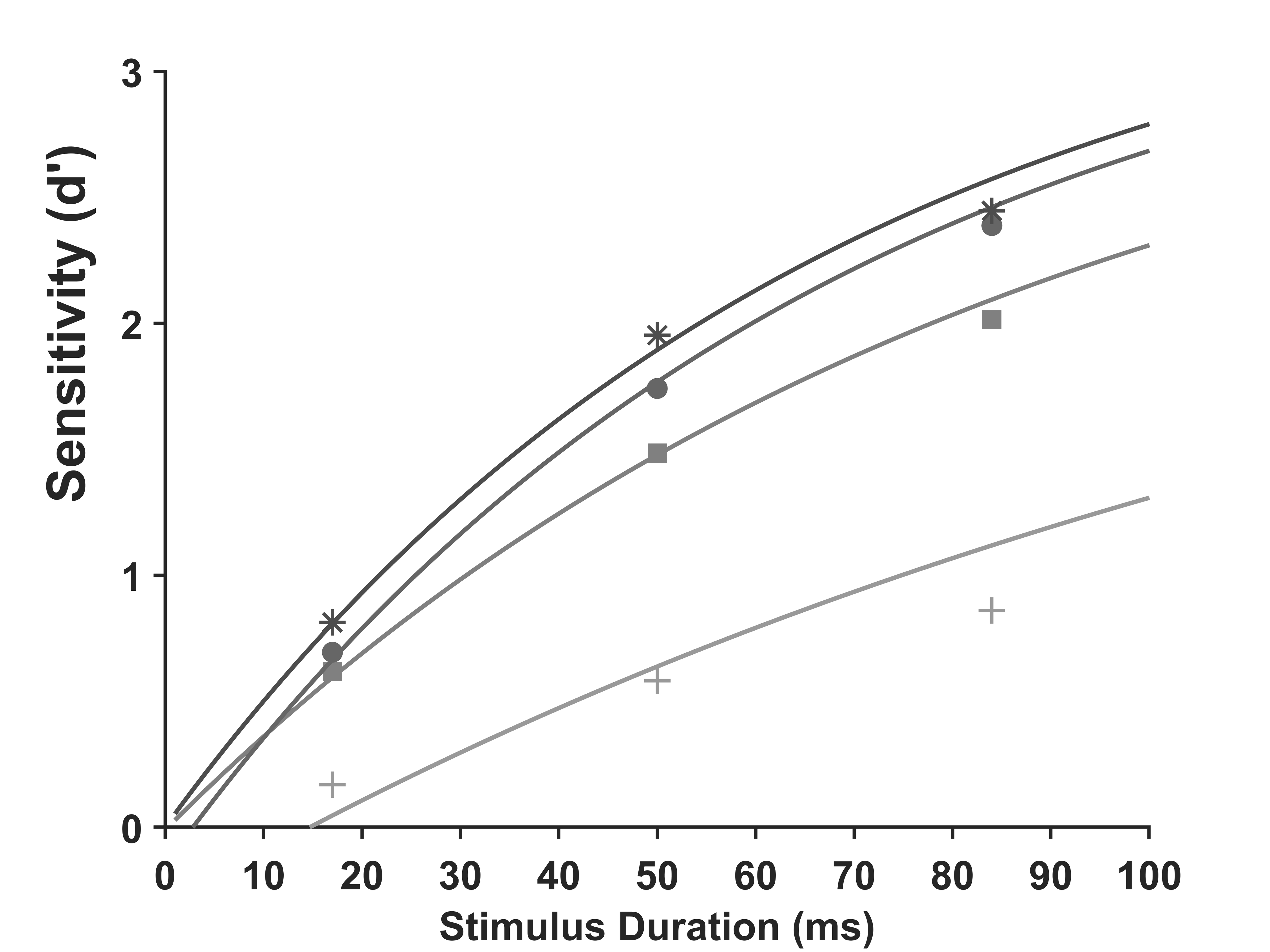

Supplement: S6 Fig — The shifted exponential function fitted to the means sensitivity data for evaluation sessions 1 (+). 4 (■), 7 (●) and 10 (*), corresponding to no training and low, medium and high training, respectively. The intercept of the curve with the 0 in the y axis (d’) correspond to the estimated onset of stimulus information extraction. (TIF) [file pone.0330284.s006.tif]
